# Anatomical Discovery—The Ligamentum Dentis

**Published:** 1839-06

**Authors:** J. F. Flagg

**Affiliations:** 31 Winter-Street, Boston,


The Publishing Committee are happy in having an opportunity to enrich the first number of their Journal with the following article from the pen of Dr. Flagg, on the subject of the *Dental Ligament*, which has been a topic of some discussion, of late, in the newspapers and magazines of the day. In the hands of a writer, distinguished alike for his professional skill and intellectual attainments, and every way qualified to investigate the question of the existence of such a ligament, the subject has been carefully examined, and, we think, satisfactorily put at rest. At any rate, the pages of this Journal will be always open to a temperate discussion of this, and every other topic connected with the usefulness of our profession.

---

*From the Boston Medical and Surgical Journal, February* 27, 1839. *Vol.* 20, *No.* 3.

### ANATOMICAL DISCOVERY—THE LIGAMENTUM DENTIS.

*To the Editor of the Boston Medical and Surgical Journal.*

Dear Sir.—I have been somewhat inclined to address you on this matter of the *new ligament*, before this, as it has made much stir in Philadelphia for these three or four months past; yet on reflection I could not but view it as of too ridiculous a character to deserve serious comment in a scientific journal. But I have been in error: and the subject has been publicly introduced in the American Journal of Medical Science, by a very respectable teacher of anatomy—Dr. P. Goddard, of Philadelphia. It is therefore, now a subject not to be passed over in silence, by those who are seeking for truth, and who are willing that the whole truth, and nothing but the truth, should be told.

While on a visit to Philadelphia, in October, I heard much of the skill of a certain dentist—a Mr. Caldwell—who extracted teeth without giving pain, or causing so little that it was not worth considering; that he removed them from the Jaw with much less effort than is usually requisite, commonly with his fingers, using no instrument but a lancet or penknife to *loosen the tooth*, which he did by cutting a previously unknown *ligament* that he had discovered, and which he declared to be the main bond of attachment between the teeth and jaw, and the principal obstruction to their removal from their bony sockets.

I treated the matter as one of the thousand that are devised to gull the public, who are generally ignorant of the truth in regard to such things. I found myself, however, so much opposed by several friends who had heard of the dentist and his discovery, and who stated many cases which had been related to them of facts, that I was determined to see Mr. Caldwell, and be informed from the only source whence I could expect to be fully satisfied.

I saw Mr. C., and after spending about an hour with him, endeavoring to get definite answers to my questions, which were mostly replied to by asking me others, or reading to me portions of manuscript which he had written on the subject, but which appeared to me to have no bearing on the question of the ligament, I was obliged to leave him with much the same impression that I had entertained previous to my call.

Although Mr. C. received me with great civility, and allowed me to ask any question that I wished, he was apparently unwilling to answer them till he had stated the views of several writers with regard to the articulations of the teeth, and the anatomy of the parts connected with them, and requested to know my views concerning certain conflicting opinions. This seemed to me to be done for the purpose of discovering whether he was dealing with one who had any definite or exact knowledge on the subject; for, as we approached it more nearly, and I had obtained all the explanation I was likely to get, and said to him, "then it seems you have not any new organ or part to demonstrate which has not been known to us before, and described by those who have written on the subject, and the parts which we call fibrous membrane and gum, or periosteum and gum, you call ligament"—his reply was something like this: "Why, sir, you know the names of parts depend on the fancy of those who give them." This appeared to me like grasping at a more slender hold, and very much like what is vulgarly called backing out. Our conversation ended here, but as I was leaving the room, Mr. C. said, the part which I call the ligament is situated between the teeth, only, and it is there that I cut to loosen the one which I wish to extract.

Since this my attention has been directed to the article of Dr. Goddard above mentioned. On reading it, I could not but feel surprised at the result of Dr. G.'s researches, if he was at all acquainted previously with the minute anatomy of the parts under consideration, and with the situation of the teeth in the different stages of their formation and development. And since Dr. G. thinks he has discovered *the ligament*, I am sorry he did not think his communication worthy of a plate which would in some degree serve as an illustration, rather than send it out with a diminutive wood engraving, perfectly indefinite in its delineation in the points which should relate to his discovery.

On the ground of a long and intimate acquaintance with the anatomy of the teeth, and of recent examinations of them with a view to furnish the remarks which I am now about to make for your Journal, I am prepared to say, that there is nothing about the teeth, their bony sockets and surrounding gum, which deserves the name of *ligament*. That there is no part of the soft structure which surrounds the teeth above the edges of the alveoli (call it by what name you will,) which serves to confine them in any remarkable manner in the jaw, or which prevents, in any perceptible degree, their being detached from their firm articulation with the sockets, in the case of an attempt to extract them. That this firm articulation of a tooth with its socket, and the corresponding resistance which is presented when we endeavor to dislocate it, depend, therefore, entirely on the intimate connection of the fang with the socket, by means of a strong intervening membrane, commonly called periosteum, on the form of the fang and socket, and on the thickness and density of the bony sides of the socket. And the part which is introduced as the *new ligament*, is nothing more than that portion of the gum which covers the transverse processes of the alveoli, united with the fibres of the periosteum above mentioned, and those of the partially absorbed capsular membrane which envelopes the crown of the tooth during its formation, and until it passes through the gum, all of which are united in a line about the neck of the tooth, immediately above the edge of the socket. And this is no more than has been known for a century at least, and may be learned from the works of Hunter, Blake, Bourdet, Fox, Thos. Bell, A. Serres, and many others.

There is, indeed, in some of the domestic animals, as the cow, sheep, hog, &c., much less of the appearance of bone about the sockets of the *front* teeth, than is found about those of man, the sockets of the former being composed more of a texture resembling a tough cartilage; yet in these the fangs of the teeth are secured entirely by the intervention of the same fibrous membrane—the periosteum.

Between the human teeth, in the mature state of either the infant or adult set, there is not room for the attachment of any ligamentous structure of sufficient magnitude or power to be of any importance in securing the teeth in their sockets, as may be seen by examining the jaw at the age of three years, or of twenty-five years. And all that can be found in the place said to be occupied by the *new ligament*, can be as clearly demonstrated to belong to one tooth as to the other, yet more justly to both; and in proportion to the thickness of the gum, as much ligament can be shown on the sides of the teeth which correspond to the outer or inner arch of the jaw, as on the sides which are in apposition, notwithstanding we are informed that the *newly discovered ligament* is situated only *between* the teeth, and is attached to but one side---the back---of the next tooth. In a word, the *dental ligament* has been brought up by a change of name, instead of a real discovery of any new part, organ or texture.

But further---admitting it to exist---you would ask, what is its practical bearing in the operation of extracting the teeth? From a full and fair examination on this point, by numerous operations, I am ready to answer, that there is no perceptible difference in the looseness of a tooth, after all the soft parts have been thoroughly severed from it, be they ligament or what you please, and before the knife or lancet has touched them; provided the bony socket and membrane are in a perfect state, or under that degree of inflammation only in which they are found previous to ulceration. If, indeed, the surrounding bone is nearly or entirely absorbed, the gum and periosteum of the fangs much inflamed and thickened, as is sometimes the case when, through fear of an operation, the patient has kept a diseased tooth for months or years, the division of these soft parts will remove all obstruction, and the tooth may be lifted away with the thumb and finger. But these cases are not very common, and have nothing to do with our question.

With regard to the pain produced in the different modes of operating, any man who is in the daily practice of extracting teeth may very soon satisfy himself that cutting round them, as a general practice, is merely inflicting needless suffering; for the use of the most improved forceps, without cutting at all, will give less pain, and the tooth is thus removed by one operation instead of two, with perfect safety and more despatch. The forceps are now in very general use, and when they are accurately made to fit the teeth, and skilfully applied, they give so much less pain than the German key in any of its varied forms, that there is no longer any reasonable apology for the dentist who does not abandon the use of this instrument entirely. In the dexterous use of the forceps, therefore, I conceive, if in anything consists the excellence of Mr. Caldwell's operations, and not in his peculiar "tact in severing the ligament," as is supposed by his friend, Dr. Goddard. And I have no doubt that if Mr. C. will lay aside his lancet, and defy the strength of the *ligament*, he will give more satisfaction and less suffering to his patients than he now does.

I beg your patience while I state a few brief questions, and I will trouble you no longer on this matter.

If there is a ligament of such strength and importance confining the teeth in the manner which the discoverers would represent, why does a tooth require the same apparent effort to dislocate it, after all the soft parts connected with it have been severed to the bone, as it would before this had been done?

To what is the *ligament* attached when the edge of the alveolus is absorbed, removing thereby the point of its origin, and leaving the neck of the tooth—the part into which we are told it is so strongly inserted—standing denuded the eighth of an inch or more above the gum. And why does this tooth stand firm under such circumstances, and require about as much force to remove it, and give as much pain in the operation, as if the bone and the *new ligament* were in their natural and perfect state, but because it is wholly supported and retained by the strong articular membrane—the periosteum of the fang and its socket?

When a tooth has been fractured or has decayed below the edge of the socket, why is it as firmly retained as when its crown and neck were perfect? There is no *ligament* here. Why does it give as much pain to extract it as if it were whole? The ligament in this case has been severed.

What holds the fang of a tooth so firmly to a portion of the outer plate of the socket, which has been drawn away with it by the use of the old key-instrument. Is it the ligament? No, that has not been discovered on this side of the tooth—yet the adhesion of these parts is so great that it will commonly require the aid of some steel instrument to separate them.

If the firmness with which the teeth are fixed to the jaw does not depend, as I have already stated, on the thickness and density of the bone, the form of the fang and socket, and the intimate attachment of the articular membrane; how does it happen, when this membrane becomes inflamed and thickened, and its vascular tissue weakened, that the tooth is loosened and lifted somewhat from its berth, and may be extracted with much less force than when the parts were in a healthy state? And why is it found, as this disease increases and the union between the periosteum and fang is, perhaps, partially destroyed, that the tooth is nearly ready to drop out, or may be taken away with the slightest effort? The *ligament* is not destroyed. It has hardly begun to share in the disease of the parts; for every practitioner in dentistry, of any observation, knows that the inflammation of the periosteum commences at the extremity of the fang and extends towards the crown of the tooth.

These are questions, involving truths, the knowledge of which every dentist of much experience must possess. Perhaps the discoverers of the *new ligament* can solve them. Yours, &c.

J. F. Flagg.

31 *Winter-Street, Boston, Feb.* 21, 1839.

